# The effect of refractive error on optokinetic nystagmus

**DOI:** 10.1038/s41598-020-76865-x

**Published:** 2020-11-18

**Authors:** Soheil M. Doustkouhi, Philip R. K. Turnbull, Steven C. Dakin

**Affiliations:** 1 School of Optometry and Vision Science, The University of Auckland, 85 Park Road, Grafton, Auckland, 1023 New Zealand; 2 New Zealand National Eye Centre, The University of Auckland, Auckland, New Zealand; 3 UCL Institute of Ophthalmology, University College London, London, UK

**Keywords:** Sensory processing, Eye manifestations

## Abstract

Subjective refraction is the gold-standard for prescribing refractive correction, but its accuracy is limited by patient’s subjective judgment about their clarity of vision. We asked if an involuntary eye movement, optokinetic nystagmus (OKN), could serve as an objective measure of visual-clarity, specifically measuring the dependence of OKN—elicited by drifting spatial-frequency filtered noise—on mean spherical equivalent (MSE) refractive error. In Experiment 1 we quantified *OKN score*—a measure of consistency with stimulus-direction—for participants with different MSEs. Estimates of MSE based on OKN scores correlate well with estimates of MSE made using autorefraction (*r* = 0.878, *p* < 0.001, Bland–Altman analysis: mean difference of 0.00D (95% limits of agreement: − 0.85 to + 0.85D). In Experiment 2, we quantified the relationship between *OKN gain* (ratio of tracking eye-movement velocity to stimulus velocity) and MSEs (− 2.00, − 1.00, − 0.50, 0.00 and + 1.00D) induced with contact lenses for each participant. The mean difference between measures of MSE based on autorefraction or on OKN gain was + 0.05D (− 0.90 to + 1.01D), and the correlation of these measures across participants was *r* = 0.976, *p* < 0.001. Results indicate that MSE attenuates OKN gain so that OKN can be used as an objective proxy for patient response to select the best corrective lens.

## Introduction

Subjective refraction is the gold standard procedure for prescription of optical correction of vision^1^. There are at least two subjective components involved in the procedure: the patient’s report on the appearance of visual stimuli, and the clinician’s interpretation of that report. Following this, having established a candidate correction, optometrists then use another subjective task, letter recognition acuity (aka *visual acuity,* VA), to quantify the resulting improvement in visual function. Although VA is the gold standard measure of visual function in the clinic, it has poor test-retest reliability^2^, which in turn limits the precision of refractive assessment. In terms of repeatability, when the same practitioner performs subjective refraction on a group of patients on different days, the 95% limits of agreement for subjective refraction is ± 0.29 dioptre sphere (D)^3^. In terms of reproducibility, when different optometrists perform subjective refraction on the same patient, the 95% limits of agreement is ± 0.55 D^4^. Such variability limits the precision of prescription of corrective lenses.

Three main approaches have sought to reduce variability in subjective refraction. First, *optical methods* utilize different optical quality metrics^5,6^ such as minimising the spread of the point spread function^7^ or maximising the peak of through-focus Strehl ratio^8^ to find an objective approximation to subjective refraction^6,7^. Objective measures of refractive error made using optical methods correlate well with subjective refraction^6,7,9,10^, but do not provide information about functional vision. Second, *electrophysiological methods* such as Visual Evoked Potentials (VEP), use measurements of brain wave potentials above the occipital cortex to infer the overall response of occipital cortex to visual stimuli^11^. The VEP approach has been used for objective evaluation of VA^12–14^, but its clinical usage is limited by the need to attach electrodes to the patient’s scalp, the duration of the test, and that its reliability under clinical conditions is unknown. Third, *involuntary eye movements* made in response to patterns containing different spatial frequencies (SFs) have been used to estimate VA^15–22^. One such involuntary eye movement is *optokinetic nystagmus* (OKN), which occurs in response to a moving stimulus. It consists of slow tracking phases (in the direction of stimulus motion), interspersed with corrective saccadic eye movements in the opposite direction. Its purpose is to (partially) stabilise the stimulus on the retina.

OKN has been used to measure VA in the presence of ocular diseases and refractive error^15–21,23–25^, with a correlation with subjective VA reported to range from *r* = 0.56^17^ to greater than 0.84^22,25^. Clinically, estimates of VA made with OKN correspond closely with subjective VA, with a mean difference of -0.01 logarithm of the Minimum Angle of Resolution (logMAR; 95% CI of − 0.58 to 0.57 logMAR)^15^. Some of these studies^16,19–21,24^ have used OKN to objectively measure VA in the presence of refractive error. However, many of these studies rely on a subjective classification method—where an examiner scores the presence or absence of OKN—and none link the VA or refractive error to the strength of OKN. Further, almost all these studies induced OKN using either black and white gratings or dot stimuli. In the presence of optical defocus such stimuli are prone to aliasing that could itself induce OKN and so reduce the impact of refractive error on the optokinetic response. The only study which has directly observed the effect of refractive error on the presence or absence of OKN (using the report of illusory perception of self-motion) claims that—as long as the stimulus remains visible—adding refractive blur as high as + 16.00 D does not affect the presence of OKN^26^. Perhaps unsurprisingly then, the authors’ conclusion was that OKN is too imprecise a tool to determine refractive error.

In this paper, we report the results from two experiments. In the first, we measured how OKN varies between individuals with different mean spherical equivalent refractive errors (MSEs). In the second, we measured how changes in MSE within each individual influenced their optokinetic response. Understanding how inter- and intra-observer variability in MSE impacts on OKN is the first step towards determining if OKN can be used to measure refractive error. Such a system would be unique in using an entirely objective measure of *functional vision* to guide the prescription of refractive correction. Several elements of our approach are novel but of particular importance is our use of drifting two-dimensional SF band-pass filtered noise to elicit eye movements. This class of stimulus is particularly valuable as it is less prone to aliasing than other stimuli used to induce OKN. In the spatial domain, band-pass filtered noise is less prone to aliasing than broad-band, e.g. dot or bar patterns when viewed in the presence of induced refractive error. In the temporal domain, filtered-noise is superior to sine-wave gratings in that we can perceive faster motion (which is better at inducing OKN) in filtered noise patterns than in drifting sine-wave grating (which are prone to direction reversal at jump-sizes that exceed the quarter-cycle limit).

## Methods

### Study design

To measure the effect of refractive error on OKN, across the population and within participants, two separate experiments were conducted. Experiment 1 examined the effect of *MSE* on OKN—across individuals—where the OKN was induced by stimuli of different SFs. Specifically, we estimated MSE, for participants with a range of refractive errors, using three different techniques. The first technique used an open-field autorefractor—a standard clinical instrument that objectively estimates MSE using an image-size principle while the proximal accommodation is minimal^27^. The second technique was to measure the *spatial frequency threshold*, the highest spatial frequency that supported participants making a reliable judgment of stimulus direction. The third technique was similar to the second but used not keypresses but the *OKN score* (a measure of the consistency of OKN with the stimulus direction) to estimate stimulus direction. We then correlated these three measures, with chart-based estimates of visual acuity, across our twenty individual participants. Experiment 2 examined the effect of *inducing refractive error*—within individuals—on the strength of the optokinetic response. The strength of OKN-response was quantified using *OKN gain*—the ratio of the velocity of eye-movements made during the tracking phase of OKN to the true stimulus velocity. Different levels of refractive error were induced using a range of contact lenses. Having measured OKN gain at different levels of induced refractive error for each participant, we selected an MSE which reliably generated strong OKN and used this as our estimated MSE. We were interested in the level of agreement between such an OKN-estimate of MSE and one made using the autorefractor*.*

Both experiments were conducted on the right eye of participants, and each took place during separate visits to the Eye Clinic at the School of Optometry and Vision Science at the University of Auckland. The experimental protocol was approved by the University of Auckland Human Research Ethics Committee (Experiment 1: ref#013247, Experiment 2: ref#022625). The study complied with the Declaration of Helsinki, and written informed consent was obtained from each participant, who were free to withdraw at any stage without giving a reason.

### Experimental setup

Stimuli were presented on a 621 × 341 mm LCD monitor (Expt-1: Dell, S2817Q, USA; Expt-2: Samsung, U28D590D, South Korea) with a 3840 × 2160 × 8-bit resolution running at 60 Hz. At the one-meter test distance, the display subtended 34 × 19 degrees. Monitors were linearised in software based on measurements made with a photometer (LS100, Konica Minolta, Japan). Experiments were written in Matlab (version 2017b, MathWorks, Natick, MA) using the Psychophysics Toolbox^28^ and the Eyelink Toolbox^29^. Monocular eye movements were recorded at 500 Hz using an infrared eye-tracker (Eyelink 1000 Plus, SR Research, Ontario, Canada) in remote mode, i.e. without the use of a chin rest. Eye-tracking data were streamed to the stimulus computer over a direct ethernet connection. The eye-tracker data stream was sampled at 60 Hz, by taking the mean of the eye-tracking samples captured between each stimulus frame refresh. The light level in the study room was photopic (698 lx), with windows covered and monitors (other than the stimulus monitor) rotated away from the participant to minimise distraction.

### Participants

For both experiments, our exclusion criteria were amblyopia or presence of neurological disorders such as epilepsy. All participants presented with normal or corrected to normal vision. In Experiment 1, two participants had astigmatic refractive error higher than − 1.00 dioptre cylinder (DC; P#13: − 1.25 DC × 122° and P#16: − 2.25 DC × 19°). In Experiment 2, we did not recruit participants with an astigmatic refractive error higher than − 1.00 DC. Participants were primarily recruited from the staff and students at the University of Auckland.

#### Experiment 1

We recruited twenty participants (20–35 years old) with a distance mean spherical equivalent refractive error ranging from + 0.25 D to − 9.25 D. To reduce the interference of participants’ optical correction on eye-tracking, participants did not wear glasses during the experiment. We fitted spherical contact lenses to induce or partially correct participants’ refractive error in order to create a gradual range of MSE between + 0.25 D and − 3.00 D across participants. Six participants did not require contact lenses because their MSE was within the desired test range. In Experiment 1, we had one MSE for each participant, and the range of the participants’ VA was − 0.16 to + 0.98 logMAR.

#### Experiment 2

Twenty-five participants (19–51 years old) with a distance MSE ranging from + 0.50 D to − 6.00 D took part in Experiment 2. Each participant wore five different contact lenses to induce a known range of MSEs; therefore, we had five MSEs for each one of 25 participants.

### Stimulus

**Figure 1:**
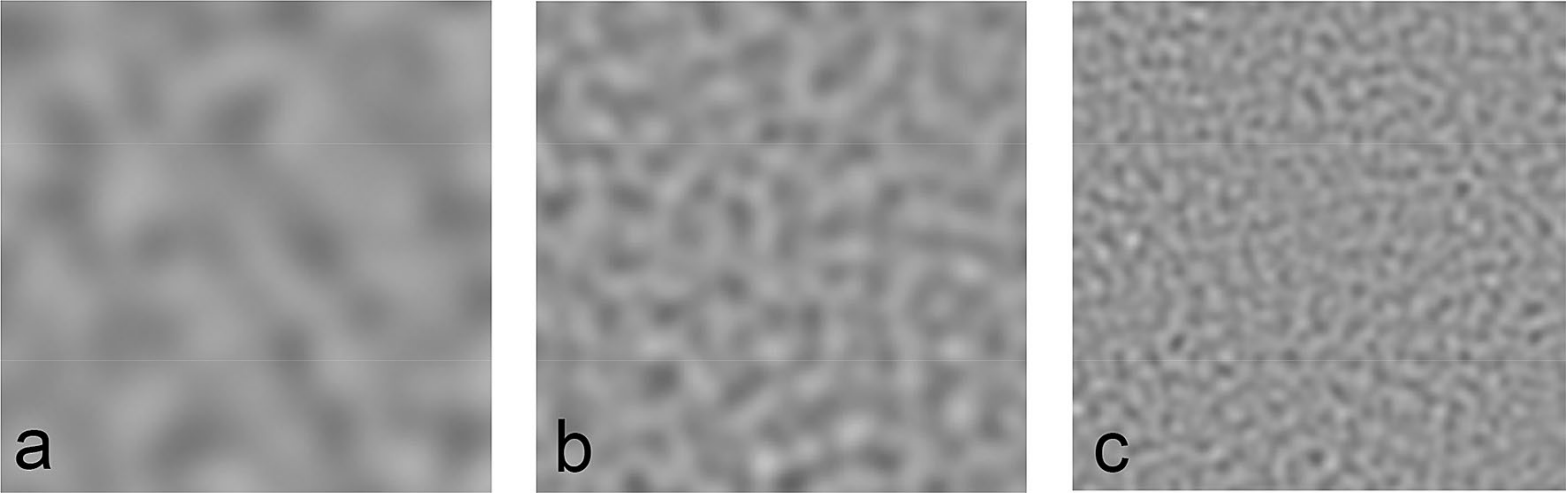
Examples of the spatial frequency filtered two-dimensional random noise movies used to elicit OKN. Here, images depict a 1 × 1 degree section of the (**a**) 4, (**b**) 8, and (**c**) 16 c/deg stimuli, all presented at 50% contrast. Note that the actual stimuli subtended 19 × 19 deg in Experiment 1, and 34 × 19 deg in Experiment 2.

Stimuli were SF filtered two-dimensional random noise patterns (Fig. 1) that were drifting horizontally (left or right) at 10 deg/s. We used a constant stimulus drift velocity of 10 deg/s, because previously it has been demonstrated that OKN reflexes elicited using this velocity are more robust^22^, independent of both the instructions given to participants^30^ and the age of participants^31^. These stimuli were generated in Matlab using two-dimensional Gaussian noise that was filtered with a log-Gabor filter to contain a narrow range of SFs in all orientations^22^. Random fluctuations in local contrast across the stimulus were minimised using a demodulation technique described previously^32^.

#### Experiment 1

The stimulus varied in SF across trials (3.8, 6.0, 9.6 or 15.2 c/deg), had a bandwidth of 0.5 octaves, and a Michelson contrast of 35%. The stimulus subtended a full-height square of 19 × 19 deg area of the screen. The participant performed 32 trials at each of four SF levels (i.e. four levels of SF × two drift-directions × 16 trials = 128 total trials).

#### Experiment 2

The stimulus varied in SF across trials (4, 8 or 16 c/deg) and had a fixed contrast of 50% for each induced refractive error. The stimulus was the size of the display, i.e. a rectangle of 34 × 19 deg. Participants underwent 16 trials for each of the three SF levels (i.e. three levels of SF × two drifting directions × eight trials × five induced refractive error conditions = 240 total trials or 48 total trials per induced refractive error). In each trial, the stimulus moved for two seconds with drift-direction (left or right) randomly stratified to ensure an equal number of leftwards and rightwards trials. To minimise any build-up of motion adaptation, a grey screen appeared for at least 1 s between trials.

### Procedure

For both experiments, we performed a nine-point eye-tracker calibration procedure before each experiment/condition. Prior to the main experiment, we ran a series of 2 s demo trials for participants who were unfamiliar with the task. Participants were instructed to maintain fixation on the centre of the screen consistent with a “stare OKN” procedure^30^. In term of instruction for participants, in “look OKN” participants are instructed to follow individual patterns of the stimulus and in “stare OKN” participants are tasked to look at the centre of the screen and to “stare straight ahead”^30^. Trials were automatically repeated when less than 80% of possible eye-tracking samples were recorded (e.g. because the participant looked away or blinked). We did not use cycloplegic agents—which prevent participants from accommodating—in order to better replicate the real-world task of subjective refraction and to avoid changes in the optical aberrations of the eye (which may affect stimulus visibility) that accompanies an increase in pupil size.

#### Experiment 1

Participants’ MSE and distance VA were measured using an open-field autorefractor (NVISION-K 5001, Shin-Nippon, Japan) and a digital Early Treatment Diabetic Retinopathy Study (ETDRS) chart presented at 6 m viewing distance. Their left eye was occluded with a patch, while their right eye viewed a series of horizontally moving patterns (leftwards or rightwards) with varying SFs at 1 m distance. Participants were tasked with indicating the stimulus direction using the computer keyboard. If the response was correct, a green disc appeared, and if the response was incorrect, a red disc appeared. Following feedback, another uniform grey screen appeared for 0.5 s before the onset of the next stimulus. The mean testing time for each participant was 6.4 min for Experiment 1.

#### Experiment 2

We fitted contact lenses to each participant to induce a known amount of MSE (D). The power of these contact lenses was calculated based on participants’ distance objective refractive error relative to the one-meter testing distance, measured using an open-field autorefractor (NVISION-K 5001, Shin-Nippon, Japan). To induce myopia, we added additional convex spherical power, and to induce hyperopia, we added additional concave spherical power to the fitted contact lenses. In total, each participant was fitted with five contact lenses: one fully corrected their refractive error, three simulated myopia (− 2.00 D, − 1.00 D and − 0.50 D), and one simulated hyperopia (+ 1.00 D). The order that these contact lenses were fitted was randomised for each participant. Their left eye was occluded with a patch, while their right eye viewed a series of horizontally moving patterns (leftwards or rightwards) with varying SFs at 1 m distance. Participants were required to indicate the stimulus direction using the computer keyboard. The colour (green or red) of a frame around the screen provided feedback on their response. We used a peripheral frame to avoid providing an explicit fixation marker for subsequent trials. Following feedback, another uniform grey screen appeared for 0.5 s before the onset of the next stimulus. The mean testing time for each participant was 20 min for Experiment 2.

### Quantifying OKN

We used two measures of optokinetic reflex. The first was *OKN score*, a classification of the sequence of eye movements made during stimulus presentation as being consistent (1) or inconsistent (0) with an optokinetic reflex in the direction of stimulus movement^22^. We also measured *OKN gain*, a continuous variable defined as the ratio of eye movement velocity during the tracking phase of OKN, to the velocity of the stimulus. If the eye moves at the same velocity as the stimulus, then the OKN gain would be + 1, if the eye did not move at all, the gain would be 0, and if the eye moved in the opposite direction to the stimulus (at the same velocity), the gain would be − 1.

Eye movement data were analysed offline after the experiment was complete. For ease of analysis, in trials with a leftward moving stimulus, eye position data were rotated 180°, so all eye movements were expressed relative to a common (0° = rightward) stimulus direction. Eye-tracking data were first filtered to remove artefacts such as blinks. We did this by removing samples where the eye-tracker was unlikely to have returned a reliable estimate of eye position indicated by the estimated instantaneous pupillary area deviating by more than 2.75 × SD from the mean pupillary area for the trial. To be conservative and allow time for the eye-tracker to reacquire the eyes, we also removed five samples (83.3 ms = 5 × 16.7 ms of the 60 Hz sampled data) from either side of any samples that were excluded on this basis. Such filtering necessarily breaks up the sequence of eyetracking data, generating sub-sequences. In order to reduce noise on estimates of eye position, we applied a second-order Savitzky–Golay filter^33^ with a frame length of five samples, to data within each sub-sequence. We used the derivative of the filtered eye position as our estimate of the instantaneous velocity of the eye.

**Figure 2:**
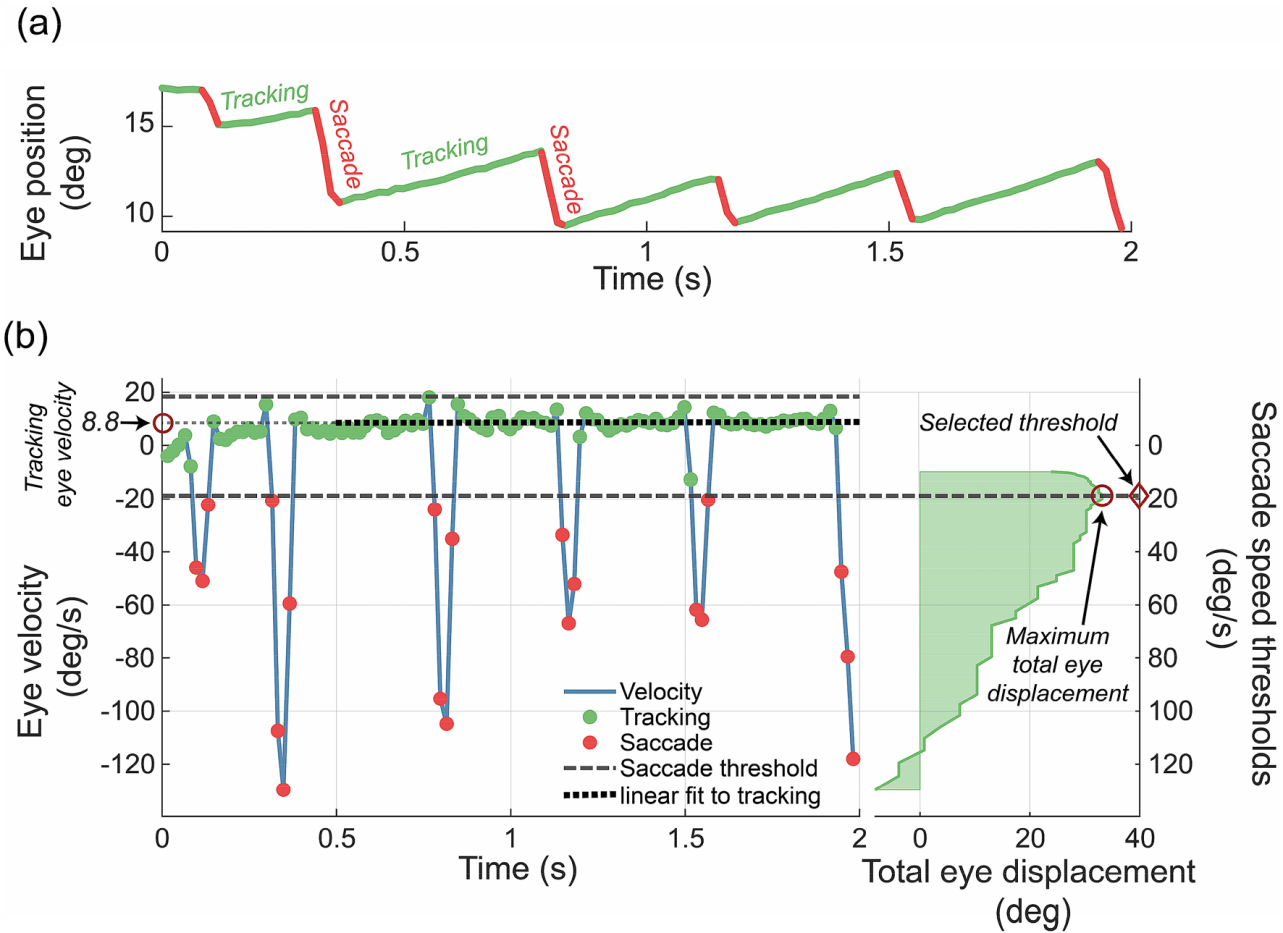
Adaptive selection of saccade threshold. (**a**) Gaze position across a 2-s trial, classified as “tracking” (green) or “saccade” (red) using the procedure illustrated below. (**b**) Estimated eye velocity (positive is similar to and negative is opposite to stimulus direction). Grey dashed lines indicate ± 1 saccadic speed threshold. Velocities whose magnitude exceeded the magnitude of the threshold were classed as saccades (red symbols). Black dotted line indicates a linear fit to the tracking data (green symbols) after the first 500 ms; here, the average eye velocity during tracking is 8.8 deg/s (i.e. the *y intercept* of the fit). The plot on the right shows an adaptive selection of saccade speed threshold from a range of candidate saccade speed thresholds. The plot shows the aggregate displacement of the eye during the trial as the sum of all tracking, minus the sum of all saccade velocities, for each candidate saccade speed threshold. The threshold selected maximised this distance. Adapted from “The Effect of Simulated Visual Field Loss on Optokinetic Nystagmus” by S.M. Doustkouhi et al., 2020, Translational Vision Science & Technology. Copyright (2020) under the Creative Commons Attribution 4.0 International license (CC BY 4.0), https://creativecommons.org/licenses/by/4.0/.

We classified each gaze point as either tracking or saccade (Fig. 2a) by selecting a threshold speed from a range of candidate “saccade speed thresholds”, which maximised the total eye displacement for that trial. The range of candidate saccadic speed thresholds for each participant on each trial spanned the stimulus speed (S, here 10 deg/s) through to S + 2 × SD of eye speed (for that trial). Eye velocity data were categorised as either *saccade* or *tracking* using each candidate saccadic speed threshold, to generate a series of candidate sets. For each candidate set, we calculated the total eye displacement by computing the sum of tracking velocities, minus the sum of all saccade velocities. The candidate saccadic speed threshold which maximised the total eye displacement for that trial was selected as the saccadic speed threshold^22^ (Fig. 2b), which was used for subsequent calculation of OKN score and OKN gain.

To calculate the OKN score, we first calculated the resultant angle from the vector sum of total eye-displacements (where each vector is comprised of the vertical and horizontal instantaneous eye-displacements) during each 2-s OKN trace. We then took the difference between this resultant angle and all possible stimulus drift directions (i.e. rightward: 0°, and leftward: 180°). The direction that had the minimum difference was selected as the OKN drift direction. We finally assigned a binary OKN score (0 or 1) based on whether the OKN drift direction was consistent (within ± 90°) with the normalised stimulus drift direction 0° (to return 1), or not (to return 0).

To calculate OKN gain the first 500 ms of each trial were excluded to allow for the rise time for OKN^34^. The average tracking velocity was calculated by fitting velocity data using a line with a slope of 0 (Fig. 2b), where the contribution of data to the fit was bisquare weighted, to reduce the influence of outliers. The y-axis intercept of the fitted line is the estimated average tracking velocity of the eye during that trial. Finally, the gain of OKN is defined as the ratio between the estimated tracking velocity and the velocity of the stimulus.

### Statistical analysis

Analyses were performed using Matlab (version 2019a, The MathWorks, Natick, MA, USA). For both experiments, we used open-field autorefractor MSEs as a reference. The reason for this choice was, while subjective refraction is the gold-standard for prescribing optical correction, objective refraction methods have the best test-retest reliability. Moreover, it has been demonstrated that an open-field autorefractor (NVISION-K 5001, Shin-Nippon, Japan; used in this study), has the best agreement with subjective refraction compared with other objective autorefraction methods^35^.

#### Experiment 1

**Figure 3:**
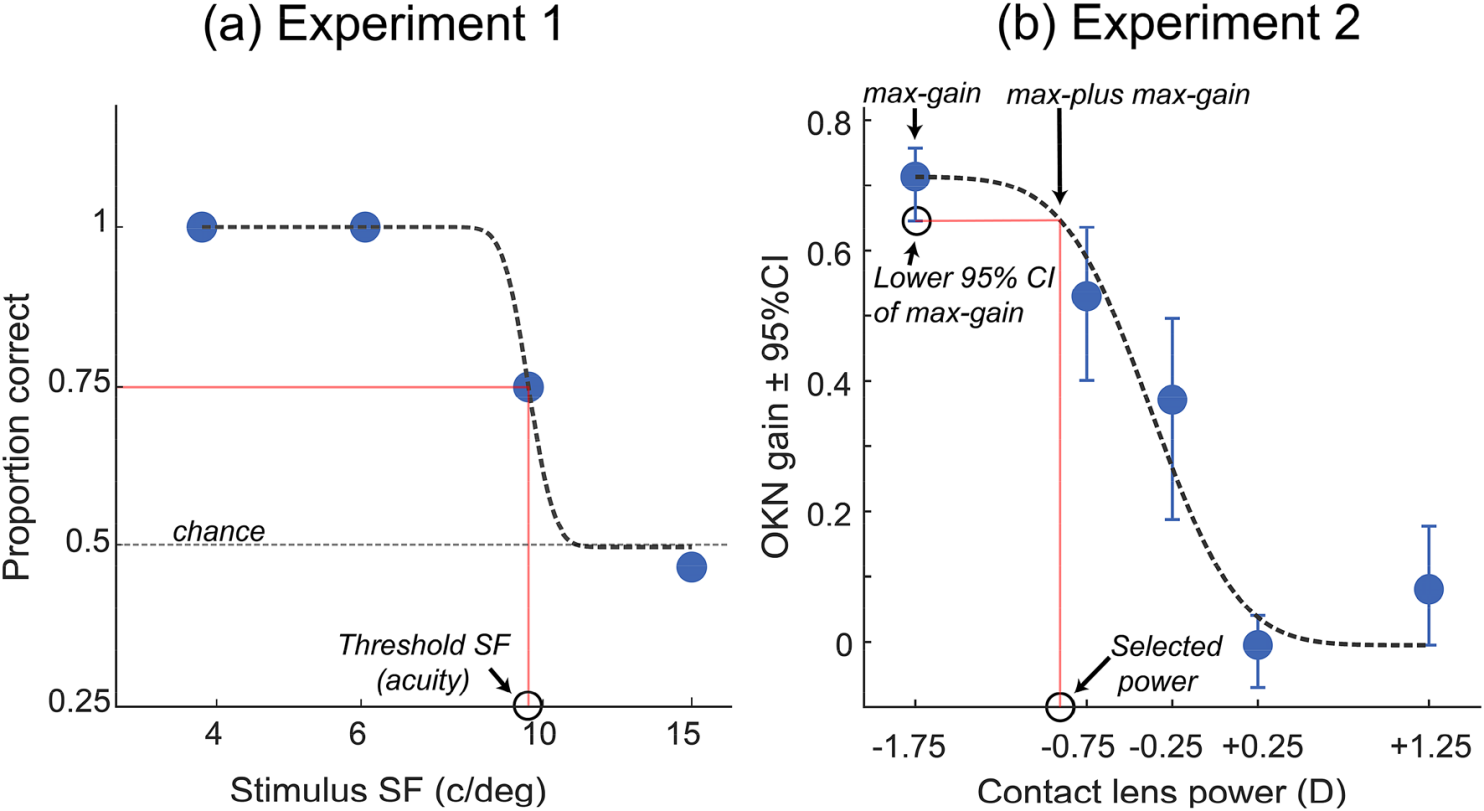
(**a**) Psychometric function fitted to the proportion correct performance based on OKN scores from Expt 1. (**b**) Mean OKN gain (blue discs) plot against the power of worn lens (D) for participant P18 in Expt. 2. Error bars are the 95% confidence intervals (CI) calculated using a bootstrap. The black dashed line is a cumulative normal fit, and the red lines show the lens power which generates a gain equal to the lower 95% CI of the maximum OKN gain. Here a lens power of − 0.90 D was predicted to generate an OKN gain equal to the lower 95% CI on the maximum gain—elicited by − 1.75 D—and so would be selected as the final MSE.

The main outcome measure in Experiment 1 was OKN score (correct or incorrect) based on consistency with stimulus drift direction. In separate procedures we fit psychometric functions to the proportion of stimuli whose direction was correctly classified (either by OKN or report) using a bootstrapping procedure implemented in Palamedes toolbox^36^. We used these fits to estimate *SF threshold—*the highest SF which elicited reliable discrimination based on either report or OKN, i.e. report-acuity or OKN acuity. As Fig. 3a shows, data were fit with cumulative normal psychometric functions, with unconstrained slopes and thresholds, a fixed lapse rate of 0, and a guess rate of 0.5. To calculate the variability of our estimate of SF threshold, we used a bootstrapping procedure with 1000 repetitions. We next fit a generalised linear model (GLM) to the refractive errors plotted against subjective thresholds based on OKN and report scores. We also calculated the Pearson’s correlations between different input and outcome measures in this experiment. Next, we used the GLM fits to predict the refractive error based on the SF thresholds and their 95% confidence intervals. Then we compared the association between the autorefractor MSE and the estimated MSE based on both OKN and report paradigms. We used Bland–Altman plots to compare the agreement between the measures (mean difference, 95% limits of agreement and their 95% confidence intervals).

#### Experiment 2

The main outcome measure in experiment 2 was *OKN gain*. We plotted mean OKN gain (pooled across 16 trials) against each induced refractive error for the three SFs tested (4, 8 or 16 c/deg). We calculated mean OKN gain and its 95% confidence interval (CI) pooled across all 16 trials of 8 c/deg for each participant at every five refractive levels. We used only 8 c/deg data as most of the participants showed a higher systematic change in their OKN gain in response to different MSEs. It is common practice for optometrists to select the best corrective lens by selecting the most plus (convex) lens which generates the best VA^1^. Here, to select the best corrective lens based on OKN gain, we used a similar procedure, choosing the maximum convex lens which generated an OKN gain equal to the lower 95% CI of the maximum OKN gain. Specifically, we fitted a cumulative normal psychometric functions to the mean OKN gain—fitting the data starting from the maximum point to the most plus worn lens power—with unconstrained thresholds, a fixed slope of − 2.5, a lapse rate value equal to one minus the maximum mean OKN gain for each participant, and a guess rate value equal to the minimum mean OKN gain for each participant. Using the psychometric function we could select the lens power which generated an OKN gain equal to the lower 95% CI of the maximum gain (Fig. 3b).

Experiment 2 used non-parametric statistics as a Shapiro–Wilk test rejected that the data came from a normally distributed population. We applied the Friedman test to analyse if there were any differences between OKN gains of different induced MSE levels. We used Pearson’s correlation and Bland–Altman plots to analyse the relationship between the MSE measured using the autorefractor and the MSE selected based on OKN-gain.

## Results

We measured how OKN changed in response to a change in refractive error, across (Experiment 1) and within (Experiment 2) participants.

### Experiment 1

**Figure 4:**
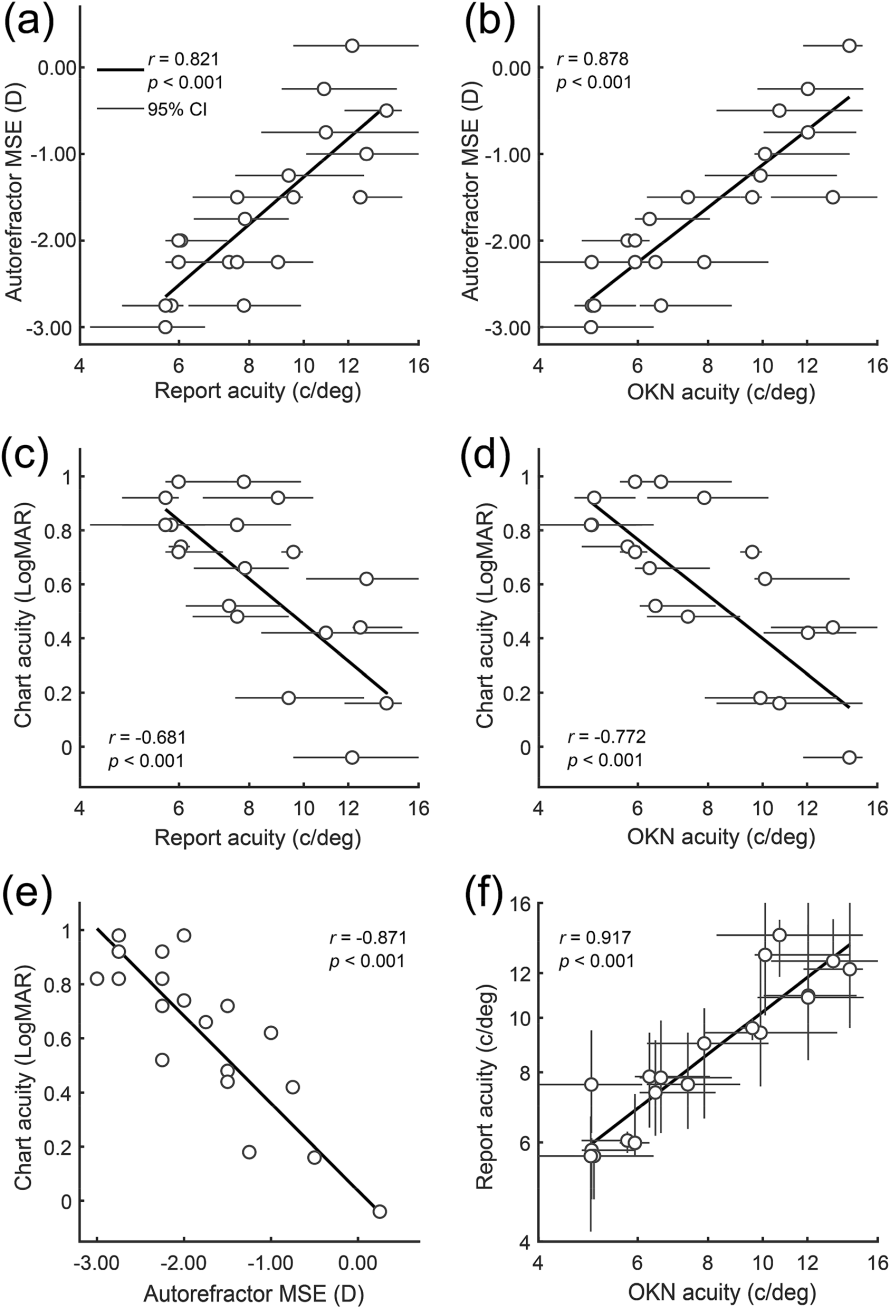
Correlations between mean spherical equivalent (MSE) refractive error measured with a autorefractor and estimates of acuity based on: ETDRS charts (*chart-acuity*), report of direction (*report-acuity*) and OKN-scores (*OKN-acuity*). Disks are the median of bootstrapped thresholds and error bars are 95% confidence intervals. The top row plots MSE against (**a**) report or (**b**) OKN acuity. The second row plots chart acuity against (**c**) report or (**d**) OKN acuity. The final row plots (**e**) chart acuity against MSE and (**f**) report acuity against OKN acuity. Note high correlation between report and OKN acuities in (**f**).

Outcome measures in Experiment 1 were the threshold SFs (acuities) and their 95% CIs, based on both keypress-report-scores (report-acuity) and OKN-consistency-scores (OKN-acuity). As Fig. 4 depicts, autorefractor MSE had correlations of *r* = 0.821, *p* < 0.001 with report-acuity (Fig. 4a); of *r* = 0.878, *p* < 0.001 with OKN-acuity (Fig. 4b); and a correlation of *r* = − 0.871, *p* < 0.001 with chart-acuity (Fig. 4e). These indicate a strong relationship between objective clinical estimates of MSE (autorefractor MSE), with three different estimates of spatial resolution: clinical chart-acuity, OKN-acuity or report-acuity. Measured chart-acuity had correlations of *r* = − 0.681, *p* < 0.001 and *r* = − 0.772, *p* < 0.001 with report-acuity and OKN-acuity, respectively (Fig. 4c,d). The correlation between report- and OKN-acuities were *r* = 0.917, *p* < 0.001 (Fig. 4f), which indicates a strong relationship between the objective OKN-acuity and the subjective report-acuity.

**Figure 5:**
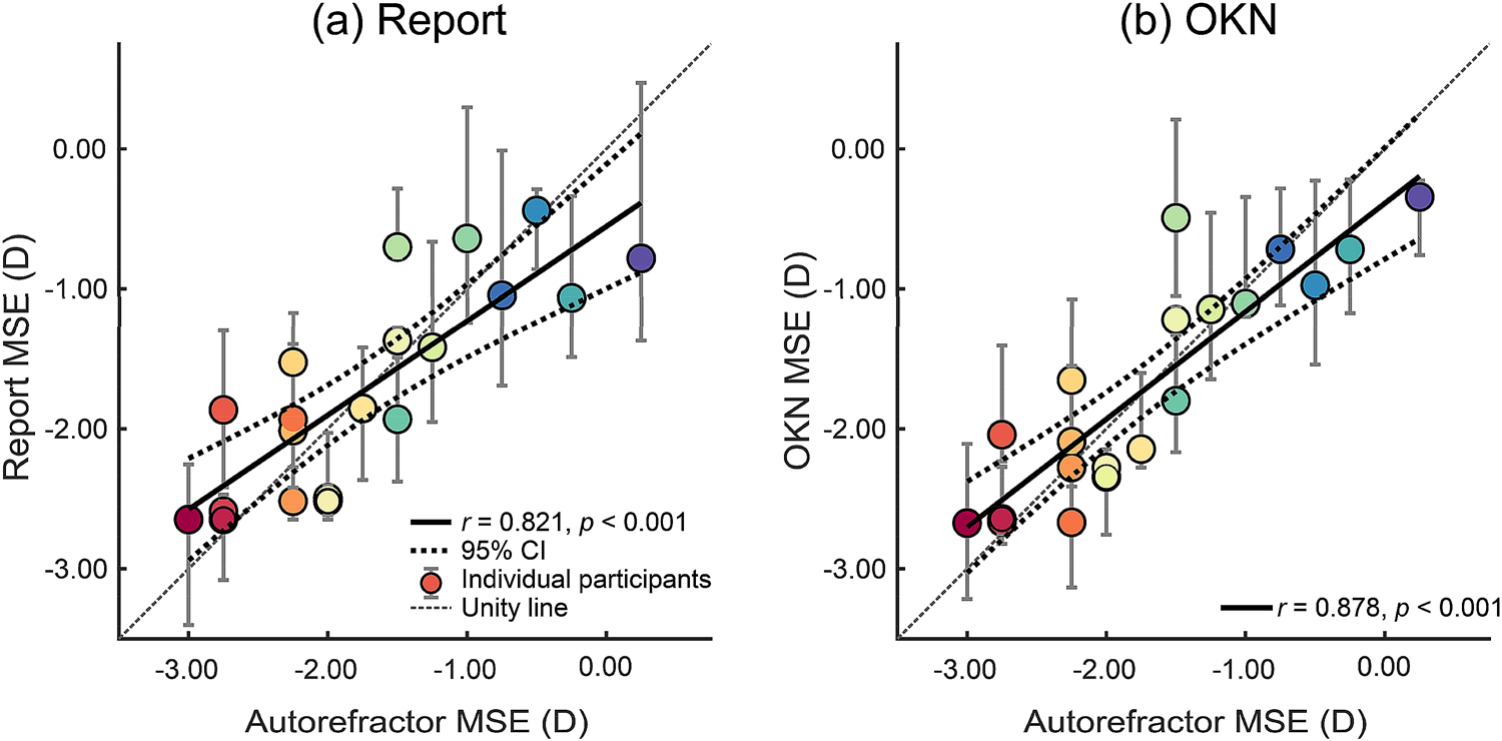
Correlation between MSE estimated by autorefractor and (**a**) MSE estimated from report scores and (**b**) MSE estimated from OKN scores. The coloured discs show individual participants and the error bars show 95% confidence intervals calculated using bootstrapping. The solid black fits show linear regressions, fitted to the plotted data, and the dashed black lines around each fit are the 95% CI of the fit.

**Figure 6:**
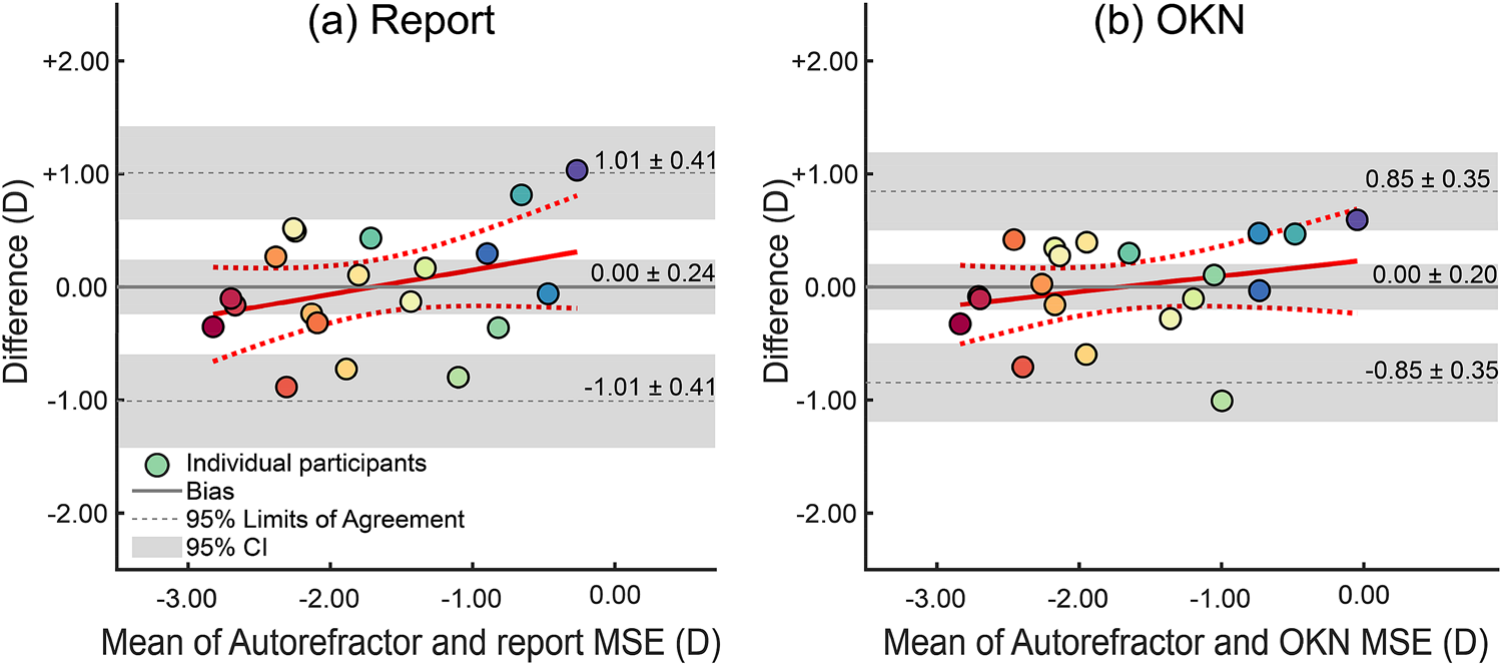
Bland–Altman plots between MSE estimated using an autorefractor and MSE estimated using (**a**) report scores and (**b**) OKN scores. The coloured discs show individual participants; thick grey lines the mean difference or bias and thin grey lines the upper and lower 95% limits of agreement (shaded red areas are the 95% confidence interval of the corresponding measure). For (**a**) bias (95% CI) is 0.00 D (− 0.24 to + 0.24 D), the lower limit of agreement is − 1.01 D (− 1.42 to − 0.60 D), and the upper LoA is + 1.01 D (+ 0.60 to + 1.42 D); and for (**b**) OKN, bias (95% CI) is 0.00 D (− 0.20 to + 0.20 D), the lower LoA is − 0.85 D (− 1.19 to − 0.50 D), and the upper limit of agreement is + 0.85 D (+ 0.50 to + 1.19 D).

Figure 5 illustrates that estimates of MSE from the autorefractor (autorefractor MSE) and estimates of MSE based on report (report-MSE) and OKN (OKN-MSE) were strongly correlated, at *r* = 0.821, *p* < 0.001 and *r* = 0.878, *p* < 0.001, respectively (Fig. 5). Figure 6a shows that the mean difference (95% CI) between autorefractor-MSE and report-MSE was 0.00 D (− 0.24 to + 0.24 D), the lower limit of agreement (LoA) was − 1.01 D (− 1.42 to − 0.60 D), and the upper LoA was + 1.01 D (+ 0.60 to + 1.42 D; Fig. 6a). Figure 6b depicts that the mean deviation of autorefractor MSE from OKN-MSE was 0.00 D (− 0.20 to + 0.20 D), the lower LoA was − 0.85 D (− 1.19 to − 0.50 D), and the upper LoA was + 0.85 D (+ 0.50 to + 1.19 D; Fig. 6b). These indicate that when compared to autorefractor MSE, although both report- and OKN-MSE had a mean difference of zero, the limits of agreement for the OKN-MSE was narrower compared to the report-MSE.

### Experiment 2

**Figure 7:**
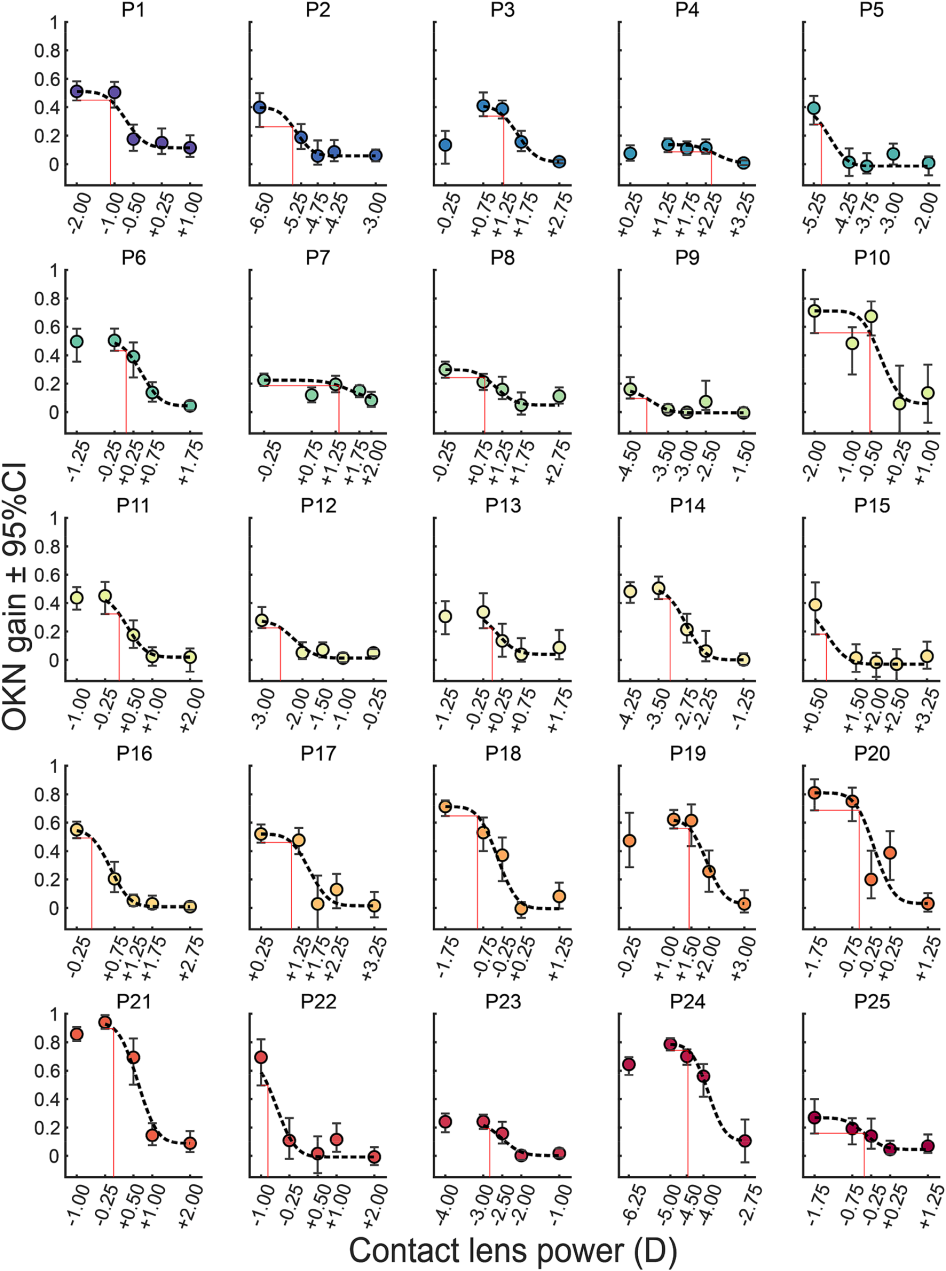
Mean OKN gain plot against lens powers worn by each participant (P1–P25). The disks are the mean OKN gain 8 c/deg spatial frequency and the error bars show 95% confidence interval (CI), calculated using 10,000 bootstrapped resamples. The dashed black lines show cumulative normal functions fitted starting from the maximum OKN gain, including all the rightward (more convex) lenses. The red lines indicate the lens power which generates an OKN gain equal to the lower 95% CI of the maximum OKN gain.

Figure 7 plots the mean OKN gain across all trials measured with an 8 c/deg stimulus against the refractive power of the worn contact lenses, for each participant. The relationship between mean OKN gains and the power of the lenses worn by each participant was captured with cumulative normal functions; *R*^2^ values of the fits had a *mean* ± *SD* of 0.856 ± 0.203 (Fig. 7). For each participant, based on their fit and the method detailed in the Statistical analysis section (see Fig. 3) we selected a best corrective lens which was the most convex corrective lens that generated a mean OKN gain as high as the lower 95% CI of the maximum OKN gain on the fit. In general, we saw higher OKN gains with contact lens powers closer to emmetropic MSEs (full refractive correction). Some participants showed a systematic change in OKN gain (e.g. P21 and P24) while others showed a non-systematic change in OKN gain (e.g. P4 and P7). Such non-systematic change in OKN would lead to the selection of a more convex corrective lens power. This indicates that although this method would be more suitable for patients with a systematic change in OKN, its inherent safety feature would prevent overcorrection of myopic patients with a non-systematic change.

There was a significant difference between OKN gains measured with different induced refractive errors for all of the three SFs used in Experiment 2 (Table 1), with higher gains seen with closer to emmetropic MSEs. The Friedman’s *χ*^2^ values in Table 1 indicate a significant main effect of refractive error on OKN gain, at all tested SFs, with a stronger main effect for the 8 c/deg stimulus.

**Table 1:** The effect of refractive error on OKN gain based on a Friedman test conducted for each SF and mean OKN gain across 4, 8 and 16 c/deg SFs.

Spatial frequency	Friedman, *χ*^2^(4, N = 25)	*P*
4 c/deg	124.11	< 0.001
8 c/deg	534.96	< 0.001
16 c/deg	15.55	0.004
Mean of 4 and 8 c/deg	506.93	< 0.001

**Figure 8:**
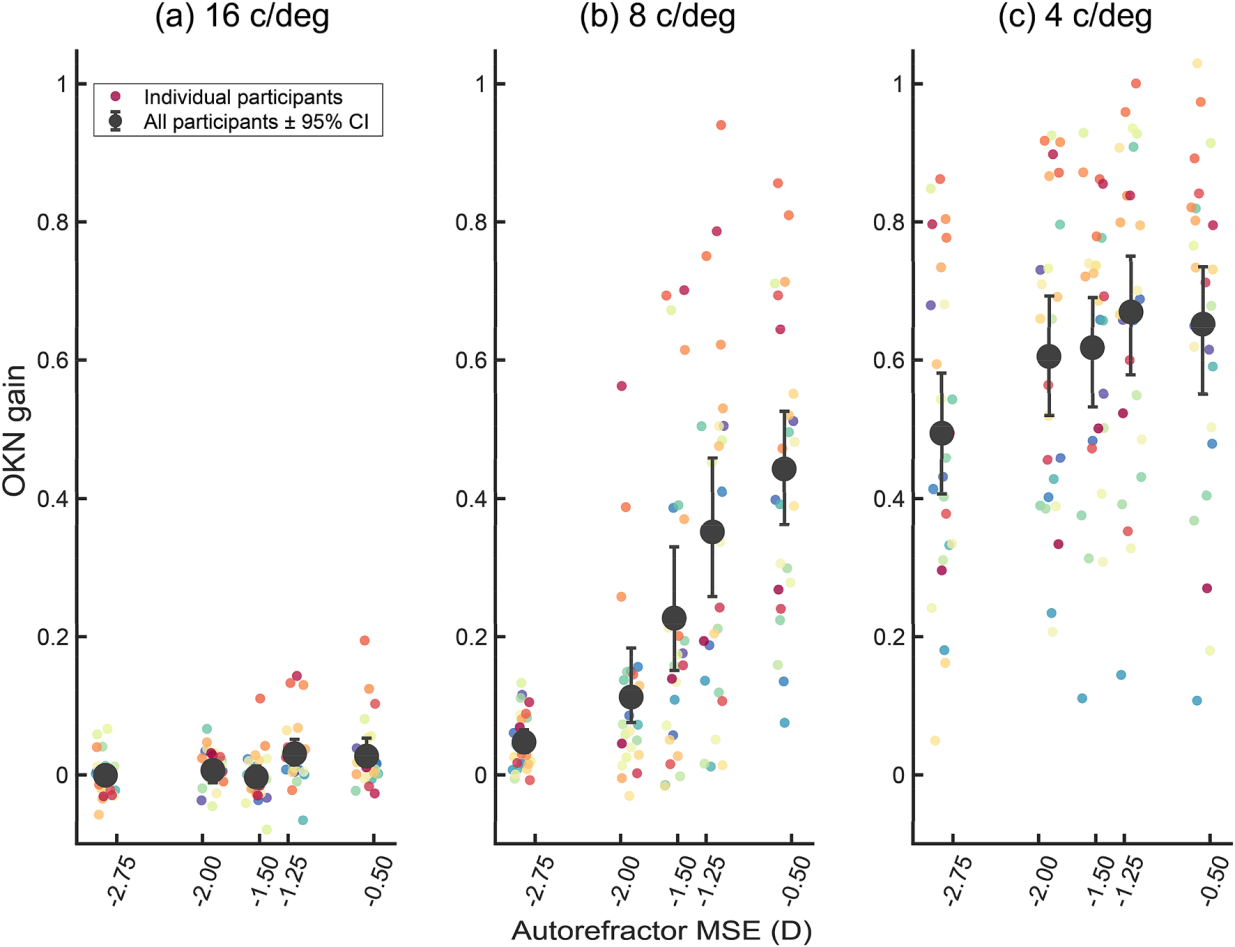
Mean OKN gain plot against mean autorefractor MSE (D) across all participants, for each of the SFs tested: (**a**) 16, (**b**) 8, and (**c**) 4 c/deg. Small coloured discs are mean OKN gains across 16 trials (colour-code based on participant number). Large grey discs are the mean OKN gain pooled across all participants. The extent of error bars shows 95% CI based on a bootstrap.

Figure 8 shows mean OKN gain (pooled across all participants) plotted against autorefractor MSE for each of the three SFs used in Experiment 2. Note the gradual increase of OKN gain as residual MSE decreases in the 8 c/deg SF condition; in contrast, there is almost no change in the OKN gain for 16 c/deg and a subtle change for 4 c/deg.

**Figure 9:**
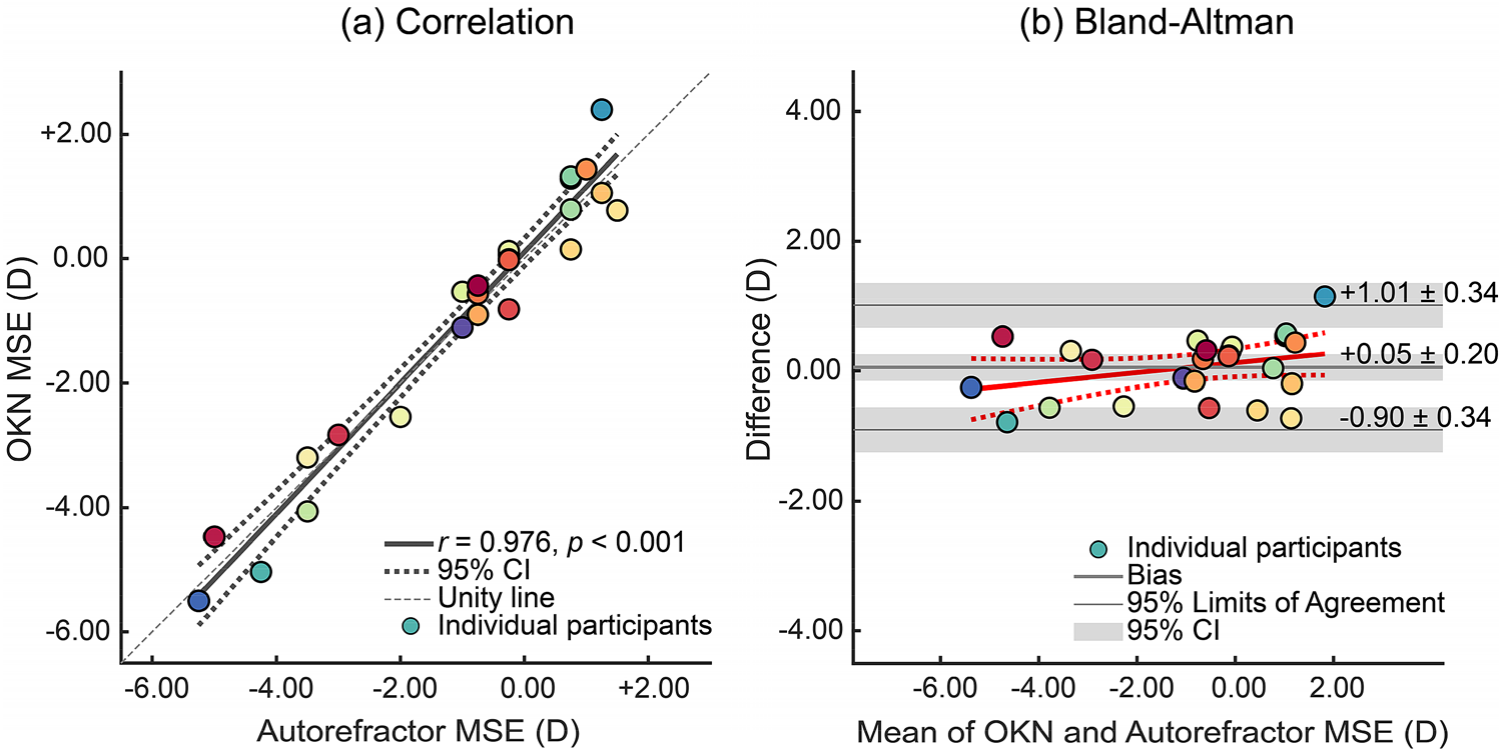
(**a**) Correlation between the MSE selected based on OKN gain and MSE estimated using an autorefractor (Spearman’s *r* = 0.976, *p* < 0.001). Confidence bounds are 95% confidence interval of the fit. The adjusted *R*-squared value of the fit is 0.950. The scatter plot is colour-coded based on participant number. (**b**) The Bland–Altman plot between MSE selected based on OKN gain and the measured MSE by autorefractor for 25 participants. The solid grey line shows the mean difference (bias) and the dashed grey lines show the limits of agreement. The shaded areas around bias, lower and upper limits of agreement show the corresponding 95% confidence intervals.

Comparison between the selected corrective lenses based on OKN gain and the objective clinical measures of MSE was made using both correlation (Fig. 9a) and Bland–Altman analysis (Fig. 9b). The Pearson’s correlation between the two measure were high (*r* = 0.976, *p* = 1.26 × 10^–16^). Mean deviation of OKN-MSE from autorefractor-MSE (95% CI) was positive, + 0.05 D (− 0.15 to + 0.26 D), the lower limit of agreement was − 0.90 D (− 1.25 to − 0.56 D), and the upper limit of agreement was + 1.01 D (+ 0.67 to + 1.35 D; Fig. 9).

## Discussion

Our results show that estimates of MSE based on OKN correlate extremely well with clinical estimates of refractive error both between individuals (in Experiment 1), and when we induced varying degrees of refractive error within individuals (in Experiment 2). The results of Experiment 1 also show that estimates of acuity based on both report and OKN scores correlated well with the subjective estimates of acuity measured with a VA chart. Critically, we report a strong correlation between subjective measures of SF threshold measured using report, with an objective measure of the SF threshold, based on OKN. These results further support the validity of using an automated objective score of OKN as a proxy for patients’ subjective response^22^.

Experiment 2 showed that OKN gain reliably decreases as the amount of induced MSE increases. These results provide the first direct evidence for the attenuating effect of refractive blur on OKN gain. However, this relationship varied over the SFs that we used; and was strongest at 8 c/deg for the range of refractive errors that we measured (− 2.00 to + 1.00 D). At refractive errors outside this range, we would expect the nominal stimulus SF to decrease as retinal blur increases. One possible explanation for these results is that increasing the amount of refractive blur would reduce the overall contrast of the perceived image. Previously, it has been demonstrated that a reduction in stimulus contrast reduces the strength of OKN^22,37^. Moreover, contrast and blur sensitivity are not equal across retina; therefore, the size of retinal input to OKN might vary for different stimulus SFs and MSEs.

In our experiments, the objective measurement of OKN served as a proxy for the clarity of visual perception in participants. In contrast, in everyday clinical practice, patients’ subjective response serves as an indicator of the quality of their visual perception. The comparison of differences-versus-mean values between these proxies for patients’ perception (i.e. objective measures of OKN in our experiment or subjective refraction in previous studies) with objective measurement of refractive error (using an autorefractor) revealed a trend consistent with previous studies. Results from Experiment 1 revealed a mean difference of 0.00 D (95% limits of agreement: − 0.85 to + 0.85 D), and the mean difference between measures in Experiment 2 was + 0.05 D (− 0.90 to + 1.01 D). Similarly, Cooper et al.^38^ reported a mean difference of + 0.19 D (− 0.74 to + 1.12 D) and Wosik et al.^35^ reported the mean differences of + 0.25 D (− 0.79 to + 1.29 D) for a closed-field autorefractor (Nidek ARK-510A) and − 0.18 D (− 1.00 to + 0.64 D) for the same open-field autorefractor used in this study (Shin-Nippon NVision-K 5001)^35^. In terms of repeatability, when the same practitioner performs subjective refraction on a group of patients on different days, the 95% LoA for subjective refraction is ± 0.29 D^3^. In terms of reproducibility, when different optometrists perform subjective refraction on the same patient, the 95% LoA is ± 0.55 D^4^. Similarly, concerning the repeatability of objective refraction, when the same practitioner performs objective refraction on a group of patients on different days, the 95% LoA was ± 0.27 D^3^. Moreover, when a group of four practitioners perform objective refraction on a group of patients on the same day, within examiner 95% LoA for different methods of objective autorefraction was ranged from ± 0.076 to ± 0.424 D, while the 95% LoA between four clinicians ranged from ± 0.149 to ± 0.291 D^10^.

In other words, repeatability studies indicate that a change of greater than ± 0.50 D is considered a clinically significant change in subjective refraction^3^. Moreover, MacKenzie^4^ demonstrated that when 40 different optometrists performed subjective refraction on a single eye (i.e. to estimate reproducibility) the results varied by a dioptre (between − 1.25 to − 0.25 D). Additionally, as Thibos, et al.^7^ demonstrated, different objective refraction methods based on wavefront aberrations have a range of mean error varying from − 0.50 to + 0.25 D, and even the most precise method, i.e. pupil fraction for slope critical pupil (PFSc), had a 95% confidence interval of ± 0.49 D. These indicate that the gold standard methods for either objective or subjective refraction have variabilities greater than 0.25 D, so our measurement is simply not fine enough to measure a subclinical refractive error with a variability lower than 0.25 D.

Previously, there have been two distinct ways to determine VA using OKN. Here, we used the “induction method”, where the highest SF which reliably elicits OKN is used to measure VA^16^. An alternative technique is the “suppression method”, where a fixation target of decreasing SF is presented until it reliably suppresses OKN^19^. Both have recorded good correlations between OKN and VA, with measures using induction ranging from *r* = 0.566^17^ to *r* > 0.84^22,25^, and with suppression OKN correlations range from *r* = 0.34, *p* = 0.064^16^ to *r* = 0.85^15,19^. Our study, with a correlation of *r* = 0.878, agrees with this existing literature. Hyon et al.^17^ report that a subjective (chart-based) measure of VA correlated better with an OKN-based measure of VA made with the suppression method (*r* = 0.83) than with the induction method (*r* = 0.57). They suggest that this finding can be explained by “central dominancy”. Central dominance theory states that although input from peripheral retina contributes to OKN, the input from the central retina plays an essential role in the induction of OKN^37,39^. However, this may not be the only reason why Hyon et al.^17^ observed a better association using the suppression method. The discrepancy between their results might be due to their use of grating stimuli of varying SF which could remain detectable beyond the human resolution limits, as a result of aliasing. In contrast, their suppression method uses a single white dot, of variable size, as a fixation marker, so that the gap between resolution and detection limits would be tighter because aliasing would not be expected. Therefore, it would better capture the actual resolution limits, and hence lead to a better correlation with subjective VA in their suppression method. In this study, we avoid this issue by using an inductive, isotropic noise pattern as the stimulus.

Both between-, and within-participants, we saw considerable variation in the amount of OKN gain with different levels of induced MSE. One source of the between participants variation in OKN gain could be the presence of a different amount of peripheral refractive error. Our stimulus covered 34 × 19 degrees of the visual field; however, we only measured foveal refraction. The resolution acuity in peripheral retina is set by sampling limits and adding refractive blur up to ± 2.00 D will only affect the detection acuity at the peripheral retina. Whereas, at the central retina, adding refractive blur would reduce both resolution and detection acuity^40^. The use of vanishing stimuli would reduce the gap between resolution and detection acuity at the central retina. A vanishing stimulus is one that disappears into the background soon after reaching the resolution limit^41^. This characteristic leads to lower threshold variability and more similar discriminability, which in turn make vanishing stimuli suitable for detecting small changes in acuity^42^. Therefore, increasing refractive blur would push the stimulus closer to the vanishing point. In other words, the use of a vanishing stimuli presentation to elicit OKN would make both central and peripheral retina sensitive to blur. It this study we measured and corrected central vision, and used global motion stimuli to measure the effect of blur on OKN. Previously, it has been shown that correction of peripheral refractive error improves peripheral motion detection^43^. OKN stimulation is not essentially a motion detection task; however, it has been shown that peripheral retina would also contribute to OKN gain. Therefore, in future investigations, the effect of correcting the peripheral refractive error on OKN properties could be examined. However, variation in OKN gain between people may not affect this technique. Further optimisations might include real-time OKN gain, with continuously varying refractive powers in order to optimise OKN-gain per person.

Another source of variation in OKN gain could be the variable effect of accommodation as we chose not to use cycloplegic agents, (a) to make the process of objective refraction more comparable to a clinical environment, and (b) because cycloplegic agents would dilate pupils which in turn would likely increase the effect of higher-order optical aberrations on our outcomes. Variability in accommodation—resulting from either the lead (i.e. over-accommodation) or lag (i.e. under-accommodation) of accommodation—could reduce the reliability of our OKN measure. We minimised such factors by using a 1-m viewing distance under which conditions the amount of lead or lag of accommodation would be near zero^44^.

Our study recruited participants with astigmatic errors less than − 1.00 DC, so we have limited ability to determine the effect of cylindrical refractive error, and any meridional blur would have confounded the relationship between spherical defocus and OKN. This leaves the question of the effect of astigmatic refractive error on OKN, which could be studied by looking at the interaction between stimulus movement direction and the astigmatic axis.

Our proposed method has the potential to provide clinicians with an objective tool to correct refractive error based on functional measures of vision. This would be a valuable clinical tool for refracting special populations who are traditionally challenging to correct, such as those with irregular corneas, non-cooperative or non-verbal patients, pre-verbal children, or people with severe cognitive impairment. It might also be of use for any longitudinal measures of refraction, as the consistency of our objective method would increase the reproducibility of traditional subjective refraction.

To develop a clinically applicable test, our procedure would need to be faster (current test time is 7 min). Shifting to an adaptive psychophysical method (from the method of constant stimuli used here) could reduce the test time to less than 2 min. To further shorten the test time, other optical methods, such as a telescopic refraction technique^1^, could be used to induce or correct MSEs (rather than the contact lenses used here). This combined OKN autorefractor would find the distance between the eyepiece and the objective lens that would reliably maximise OKN gain. This proposed device would require real-time OKN measurement and an eye-tracker that could measure the eye movements independent from the calibration change and the reflections from the surface of the lenses.
